# Life after Burn, Part I: Health-Related Quality of Life, Employment and Life Satisfaction

**DOI:** 10.3390/medicina58050599

**Published:** 2022-04-27

**Authors:** Maria Fernanda Hutter, Christian Smolle, Lars-Peter Kamolz

**Affiliations:** Division of Plastic, Aesthetic and Reconstructive Surgery, Department of Surgery, Medical University of Graz, 8036 Graz, Austria; christian.smolle@medunigraz.at

**Keywords:** burn injury, HRQoL, return to work, life satisfaction, consequences of burn injuries

## Abstract

*Background and Objectives*: As advances in medicine are proceeding, so are treatment goals shifting from sheer mortality rates to improving HRQoL and social reintegration after burn injury. Following this trend, we aimed to assess HRQoL, employment and life satisfaction after burn injury to gain insight on confounding factors. *Materials and Methods*: This single-center follow-up study was conducted using the SF-36 V1.0 in German and further questions evaluating employment and life satisfaction. It reached 128 adult in-patients (recall 33.0%) with former burn injuries, treated between 2012 and 2019 at the Division of Plastic, Aesthetic and Reconstructive Surgery at the University Hospital of Graz. The questionnaire outcomes were set into relation with clinical data obtained from the medical records. Statistical analysis was performed with SPSS 27.0 for Windows. *Results*: Of the 128 participants, 72.7% were male and 27.3% female. The mean age at the time of injury was 40.0 ± 15.7 years and mean %TBSA among the study population was 9.2 ± 11.0%. The male patients had sustained more extensive injuries (*p* = 0.005). However, the female patients scored significantly (*p* < 0.05) and consistently lower in all the domains of the SF-36, except for “bodily pain” (*p =* 0.061). Moreover, the female patients scored lower in all the domains of life satisfaction, although significant differences were only found in the domains of fulfillment (*p* = 0.050) and mental wellbeing (*p* = 0.015). Furthermore, employment status differed significantly between the male and female patients before as well as after the burn injury. Proportionally less women were employed at both time points. Overall, unemployment had declined. *Conclusions*: Life satisfaction after burn injury in this study cohort seems to be good. Return to work has shown a promising trend. Strikingly, HRQoL and life satisfaction were lower in women after burn injury. Further research on the reasons for this gender discrepancy might improve HRQoL and life satisfaction after burns.

## 1. Introduction

As advances in medicine are proceeding, treatment goals start to shift from sheer mortality rates to improving the health-related quality of life (HRQoL) of patients after burn injuries, as well as the return to everyday life and restoring psychological balance [1]. HRQoL is a concept that aims to depict the patient’s perceptions of physical, mental and social wellbeing and sets them in relation to different personal and environmental factors that influence health [2]. Reaching this goal can be challenging for patients and their relatives as rehabilitation can take up to two years and necessitate correctional surgeries, which can additionally put a mental strain on the patients [3].

In order to be able to measure HRQoL, different tools for assessment have been developed and the short- and long-term outcomes of burn victims are increasingly being studied. The most commonly used tools are the Burn Specific Health Scale Brief (BSHS), the Medical Outcomes Survey Short Form-36 (SF-36) and the EuroQol (EQ-5D) [2].

Some studies have already investigated some factors influencing HRQoL after burn injuries. Besides the extent of the initial burn injury [4], not only physical sequelae, such as persistent bodily pain [5], affect HRQoL but also the patient’s own resources, such as social background, previous medical and psychiatric history or his or her own coping strategies [6,7,8].

Employment post-burn-injury plays an important part for the return to a normal everyday life as a job might give the person a routine and purpose. Most studies have followed patients for up to two years after the burn injury and showed return to work rates between 66 and 79.7% [9,10,11,12,13,14].

To our knowledge, life satisfaction after burn injuries has rarely been assessed so far. Three studies [15,16,17] have examined satisfaction with life using the Satisfaction With Life Scale (SWLS), a five-item survey developed by Diener et al. in 1985 assessing global life satisfaction with questions that are open to interpretation. We have defined life satisfaction as being able to live an autonomous and self-determined life, feeling needed in everyday life, having a fulfilled employment or retirement, enjoyable leisure activities and being content with one’s appearance, physical functioning and mental wellbeing.

Burn injuries can have a huge impact on the HRQoL, employment situation and life satisfaction of the individual who suffered a burn injury. Our study aimed to investigate confounders of HRQoL measured with the SF-36, employment and life satisfaction after burn injuries. The hypothesis was that the extent and severity of the burn injury as well as the injury site, the age at which the burn occurred, preexisting co-morbidities and need of intensive care treatment would correlate negatively with HRQoL, employment situation and life satisfaction after the burn injury, whereas time from accident would correlate positively with both.

## 2. Materials and Methods

### 2.1. Study Design

This study was a single-center follow-up study of the self-reported HRQoL, employment situation and life satisfaction after burn injuries of in-patients treated at the Division of Plastic, Aesthetic and Reconstructive Surgery, Department of Surgery at the University Hospital of Graz between 1 January 2012 and 31 December 2019. The survey was conducted between November 2020 and May 2021.

### 2.2. Study Endpoints

The primary study endpoints were the self-reported HRQoL measured with the SF-36 V1.0 in German at the time of questioning, employment status up to the injury and at the time of questioning as well as life satisfaction at the time of questioning.

Secondary endpoints were the relation of the results of the SF-36, employment status and life satisfaction to clinical data, injury characteristics as well as therapy-related parameters and level of education.

### 2.3. Patient Collective, Inclusion and Exclusion Criteria

All patients with former burn injuries, regardless of mechanism, affected % total body surface area (%TBSA) and depth of the injury, age ≥ 18 at the time of injury and able to fill out the questionnaire (i.e., no severe cognitive impairment) who were treated as in-patients at the Division of Plastic, Aesthetic and Reconstructive Surgery at the Department of Surgery at the University Hospital of Graz between 2012 and 2019 were deemed eligible for the study. Importantly, patients had to have an Austrian phone number. Patients were contacted by telephone, informed about the study and asked whether they wanted to participate in it. If they gave their consent, they received an email with the study information and a written informed consent, which they were asked to sign and return by email. Patients without access to the internet were sent a letter by post.

### 2.4. Collected Clinical Data

Based on the medical records, following clinical data were collected: age at time of injury, gender, date of injury, injury mechanism, extent of injury (in %TBSA), injured site (if applicable), days at intensive care unit, days of mechanical ventilation, need for tracheotomy, number of operations until discharge, length of hospital stay (=LOS) and comorbidities (diabetes mellitus, arterial hypertension, ischemic heart disease, cerebrovascular disease, peripheral vascular disease, psychiatric disorders, history of substance abuse (alcohol, nicotine, drugs)).

### 2.5. Questionnaire

For the assessment of HRQoL, the Short-Form-36 Health Survey (SF-36) V1.0 was used since it is a well-recognized multidimensional tool for assessment of patient-reported HRQoL and has also been validated for outcomes in burn survivors [18]. The SF-36 exists among 170 languages and also in a validated German version. Version V1.0 was used for this survey [19,20,21,22].

The SF-36 encompasses eight sections (vitality, physical functioning, bodily pain, general health perceptions, physical role functioning, emotional role functioning, social role functioning, mental health). The weighted sums of these eight scales are directly transformed into 0–100 scales based on the assumption that each section carries equal weight. The lower the score, the more disability [20,23].

Following the SF-36, the interview was complemented with further questions consisting of multi-alternative, single-choice questions. Herein, level of education, employment until the burn injury and at the time of questioning were queried. This was followed by questions about their life satisfaction (see Table 1).

The answers to these questions were not transferred into a comparable scoring system but individually assessed and set into relation with clinical data.

The survey was carried out via telephone. The questions were read to the participants and their answers were noted.

### 2.6. Statistical Analysis

The statistical analysis was performed with the statistical program SPSS 27.0 for Windows. The correlations were measured by chi-squared test, t-test, Mann–Whitney U-test, Spearman correlation and regression analysis. The relative influence of different parameters on the test results was determined by linear regression or binary logistic regression analysis, and *p*-values < 0.05 were considered statistically significant.

## 3. Results

### 3.1. Included Patients

**Figure 1:**
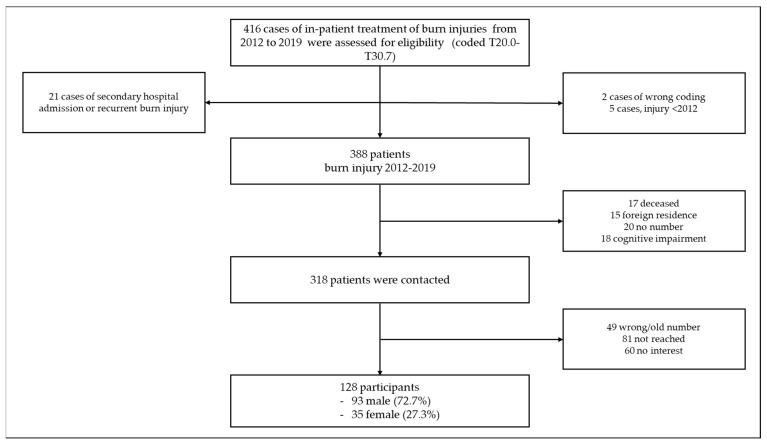
Flowchart outlining the selection of participants.

Between 1 January 2012 and 31 December 2019, 416 in-patient cases treated for burn injuries (coded by ICD-10 T20.0-T30.7) at the Division of Plastic, Aesthetic and Reconstructive Surgery at the Department of Surgery of the University Hospital of Graz were assessed for eligibility. In total, 128 patients (recall 33.0%) were willing to participate in the study. At the time of inquiry, the mean time since injury was 61.1 ± 25.4 months and did not differ significantly between both genders. It ranged between 17 and 107 months. For details, see the flowchart (Figure 1).

### 3.2. Demography

Of the 128 participants, 93 (72.7%) were male and 35 (27.3%) female. The mean age at the time of injury was 40.0 ± 15.7 years and at the time of inquiry 45.1 ± 16.2 years. The female patients were slightly older at the time of injury (41.2 ± 16.3 years) and at the time of inquiry (46.1 ± 16.4 years) versus the male patients (39.5 ± 15.6 years at injury; 44.7 ± 16.2 years at inquiry), although not significantly. The age range was 18 to 73 years at the time of injury and 21 to 79 at the time of inquiry.

The highest level of education was apprenticeship for most of the participants (*n* = 82, 64.1%). Nineteen (14.8%) participants had a university degree, 15 (11.7%) ended their scholarly education after middle school and 12 (9.4%) participants had a general qualification for university entrance at the time of inquiry. There were no significant gender differences throughout the levels of education.

Documented pre-existing medical conditions were found in about one third of the participants (*n* = 41, 32.0%). The most common disease was arterial hypertension, affecting 19 patients (22% of the female participants and 11.8% of the male participants). The second most common pre-existing condition was a history of psychiatric disease (*n* = 8), predominantly affecting female patients (17.1% vs. 2.2% in males, *p* = 0.002). Further, history of substance abuse was also associated with a higher likelihood of female gender (*p* = 0.020), as was history of alcohol abuse (*p* = 0.030; for details, see Table 2).

### 3.3. Injury Characteristics

Table 3 provides an overview of the injury characteristics overall and per gender.

The overall mean TBSA was 9.2 ± 11.0% and was significantly higher in male patients (*p*-value 0.005).

Relatively, burn injuries to the lower extremity were more common in female patients (65.7% versus 48.4% of each group), whereas, in men, the upper extremity (69.9% versus 48.6%), the hands (47.3% versus 25.7%) and the face and neck (55.9% versus 31.4%) were more often affected. Five (3.9%) patients, all male, sustained inhalation injuries in addition to external burns (Table 3).

**Figure 2:**
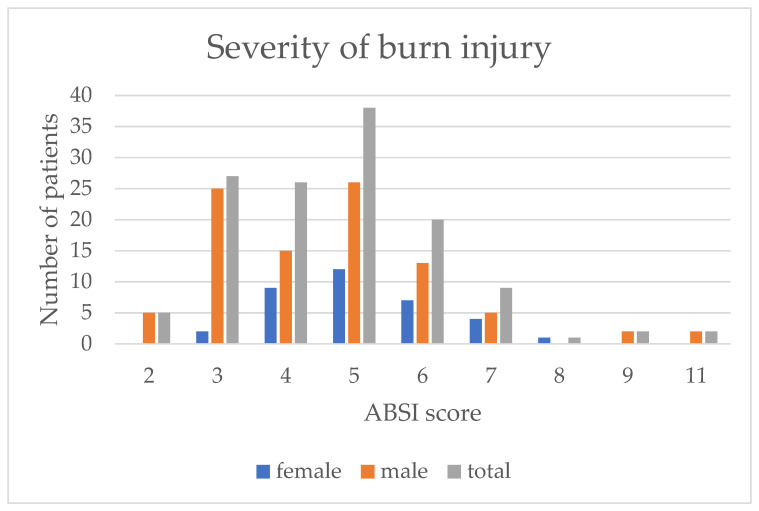
Severity of burn injuries among participants based on the ABSI score by gender.

The severity of the burn injuries was depicted by the ABSI score. The data showed that the median ABSI was 5 in all the cohorts, with only the IQR differing between female (IQR 4–6), male patients (IQR 3–6) as well as for the entire study population together (IQR 3.5–6; see Figure 2).

### 3.4. Treatment

The mean length of stay (LOS) in the hospital was 15.5 ± 16.4 days. Twenty-four patients required intensive care treatment at the ICU (twenty-two male and two female) and stayed there on average for 12.0 days (range 1–47). Of those, 21 had to be intubated and spent a mean of 8.7 days on the ventilator (range 1–37). Seven patients required a tracheotomy (five male and two female). The majority of the patients (*n* = 91, 71.1%) required at least one surgery, with a mean number of surgeries of 1.1 (range 0–7).

### 3.5. Health-Related Quality of Life

**Figure 3:**
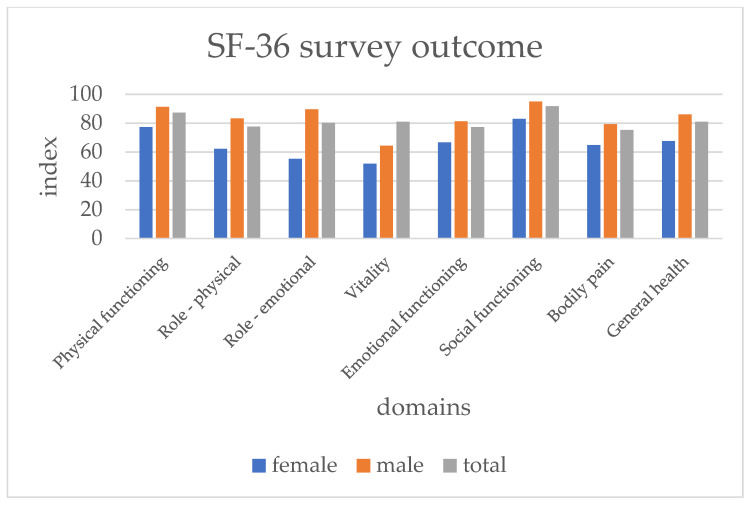
Mean of SF-36 domains by gender.

First, the answers of the SF-36 were analyzed separately by gender. Female patients scored significantly and consistently lower in all the domains, except for the domain bodily pain (see Figure 3 and Table 4).

Next, a stepwise linear regression analysis was performed to detect the variables that influenced individual domains of the SF-36. The suspected variables were clinical data (age at injury, gender, time since injury, %TBSA, third-degree burn injury, burn of hands, burn of genitals, burn of face or neck, inhalation injury and LOS).

The physical functioning domain correlated positively with male gender (*p* < 0.001, coefficient 16.86), negatively with days of LOS (*p* = 0.000, coefficient −0.55), also negatively with third-degree burn injury (*p* = 0.023, coefficient −7.40) and age at injury (*p* = 0.037, coefficient −0.21). Role limitations due to physical health showed a significant correlation with LOS (*p* < 0.001, coefficient −0.75) and male gender (*p* = 0.001, coefficient 24.19). Age at injury also had a negative correlation; however, this was not significant (*p* = 0.077). For the domain role limitations due to emotional health, the strongest confounders were male gender (*p* < 0.001, coefficient 36.77) and third-degree burn injury (*p* = 0.002, coefficient −20.00). Vitality, as well, correlated positively with male gender (*p* < 0.001, coefficient 13.48) and inversely with third-degree burn injury (*p* = 0.028, coefficient −7.42). The same was observed for the domain emotional wellbeing (male gender, *p* < 0.001, coefficient 15.85; third-degree burn *p* = 0.002, coefficient −8.95). Social functioning had a significant correlation with male gender only (*p* = 0.010, coefficient 12.12). Significantly correlating variables for the domain pain were LOS (*p* = 0.001, coefficient −0.81), male gender (*p* = 0.010, coefficient 16.63) and TBSA (*p* = 0.024, coefficient −0.83). Affection of the hands also influenced pain, but not significantly (*p* = 0.060, coefficient −11.29). At last, general health correlated significantly with male gender (*p* < 0.001, coefficient 20.31), third-degree burn injury (*p* = 0.014, coefficient −10.53) and LOS (*p* = 0.005, coefficient −0.41). Inhalation injury correlated with a *p*-value of 0.072 (coefficient 20.71).

### 3.6. Employment Situation

The majority of the participants (*n* = 95, 74.2%) were employed before they sustained the burn injury, 20 (15.6%) had already retired and 13 (10.2%) participants were unemployed. Students were counted as unemployed. There was a significant difference in employment status between the male and female patients (*p* = 0.003). More male participants than female participants were employed at the time of injury (*n* = 77, 81.1% vs. *n* = 18, 51.4%). Relatively, more female (*n* = 9, 25.7%) than male participants (*n* = 11, 11.8%) were retired at the time of injury. Eight female patients (22.9%) and five male patients (5.4%) were unemployed at the time of injury.

After the burn injury, 57.1% of the female participants (*n* = 20) and 72.0% of the male participants (*n* = 67) were able to return to their pre-burn jobs with the same number of working hours. Further, 2.9% of the females and 7.5% of the males changed their jobs but continued with the same working hours; 2.2% of the male participants stayed in the same job but reduced their working hours; 2.9% of the female and 4.3% of the male participants changed their jobs and working hours; 17.1% of the females and 2.2% of the males reintegrated into working life without having a job before the injury; 11.4% of the female patients and 8.6% of the male patients had retired between the time of injury and inquiry; 5.7% of the women and 1.1% of the men had lost their job. One woman and two men continued unemployed. The differences in change in employment status were not significant between both genders (*p* = 0.898).

In total, eighty-eight (68.8%) of the participants were employed at the time of inquiry, thirty-two (25.0%) had retired and eight (6.3%) were unemployed. At this time point, there was a difference in employment between male and female patients as well (*p* = 0.023). The number of employed females remained stable, while the number of employed males declined to 70 (75.3%). The rates of retirees increased in both groups, to 37.1% (+4) among the female participants and 20.4% (+8) among the male participants. Unemployment declined in both groups, overall—3.9%, −11.5% for female participants and −1.1% for male participants. Further, 6.3% of the patients (*n* = 8; six females and two males) who were unemployed before the burn injury got a job after recovery of the burn trauma. For detailed data, see Table 5.

A stepwise regression analysis showed that being employed after the burn injury highly correlated with being employed before the burn injury (coefficient 0.54, *p* < 0.001), age at injury (coefficient 0.02, *p* < 0.001) and also lower level of education (coefficient −0.22, *p* = 0.012).

### 3.7. Life Satisfaction

The outcomes for autonomy (97.8% vs. 94.3%), being needed (95.7% vs. 94.3%), fulfillment (90.3% vs. 77.1%) and joy (94.6% vs. 85.7%) were consistently stated better by male participants than females. However, significant differences were only found in the domains fulfillment (*p* = 0.050) and mental wellbeing (*p* = 0.015; see Table 6).

Hereafter, a logistic regression analysis was performed to detect confounders. For autonomy, no connection to clinical data could be found. Being needed has shown to positively correlate with longer LOS (OR 0.95, *p* = 0.004). The same was observed for fulfillment (OR 0.96, *p* = 0.036). Months since injury was found to be a negative confounder for joy (OR 0.97, *p* = 0.030). The test further showed that the wish to change aesthetics correlated with longer LOS (OR 1.05, *p* = 0.007), younger age at injury (OR 0.96, *p* = 0.018), male gender (OR 0.36, *p* = 0.045) and third-degree burn injury (OR 2.97, *p* = 0.046). The desire for better body functionality correlated with younger age at injury (OR 0.94, *p* < 0.001), burn injury of the face or neck (OR 0.29, *p* = 0.011) and burn injury of the hands (OR 3.05, *p* = 0.021). Confounders for the desire to change mental wellbeing were male gender (OR 0.25, *p* = 0.007) and third-degree burn injury (OR 4.06, *p* = 0.010).

## 4. Discussion

The novelty of the present study is that patients’ rehabilitation after a burn injury was not only assessed using the well-established SF-36 survey but also with assessment of employment and life satisfaction. These questions aimed to obtain insight into the patients’ self-evaluation of their life situation.

The present study assessed the HRQoL, employment situation and life satisfaction between 17 and 107 months after the burn injury of about a third of all the patients treated at the Division of Plastic, Aesthetic and Reconstructive Surgery at the Department of Surgery of the University Hospital of Graz in the study period, which is a high proportion.

The hypothesis was that the extent and severity of the burn injury as well as the injury site, the age at which the burn occurred, preexisting co-morbidities and need of intensive care treatment would correlate negatively with the HRQoL, employment status and life satisfaction after the burn injury and that it is proportional to the time between the incident and questioning.

### 4.1. Study Population

In this study population, the majority of the participants were male (72.7%), which aligns with several other studies considering worldwide burn injury trends showing a male predominance of about 50% more male burn injury patients in adult burn injuries [24,25,26]. A significant difference between those two groups was observed regarding co-morbidities. Most assessed pre-existing conditions affected only a paucity of the study participants, which might have been rooted in the relatively young age of the participants (40.0 ± 15.7 years). A history of psychiatric disease, substance abuse and alcohol abuse, however, was observed in 9.4% of the participants and had been reported in the medical records significantly more often within the female cohort. Psychiatric disease had been documented in 17.1% of the female participants compared to 2.2% of the male participants. However, there is a certain blurriness in these data as mental health was not assessed at admission nor in the course of rehabilitation, which could have been impaired due to the trauma. Dyster-Aas et al. have observed that two-thirds of their burn patients developed at least one psychiatric diagnosis during the course of their lives; however, a great part had already suffered from a psychiatric disease before the burn injury [27]. Moreover, all pre-existing conditions were only assessed by checking past medical records from the time of injury and not at the time of interview. Therefore, it was not possible to investigate the influence of pre-existing conditions on HRQoL, employment status and life satisfaction.

### 4.2. Health-Related Quality of Life

One of the main findings was that HRQoL and life satisfaction are predominantly influenced by gender. The female patients scored lower in all the domains of the SF-36 survey, although, significantly, only in seven out of the eight domains, excepting for the domain bodily pain, which is especially interesting as, in the present study, women sustained significantly less extensive burn injuries. Moreover, Spronk et al. have observed in two recent studies that female patients, together with patients who had a longer LOS and had sustained more extensive burn injuries, suffered from lower HRQoL post-burn [28] and that, although weaker than burn severity, psychological factors and unemployment, female gender seems to be a negative predictor for HRQoL [29]. Furthermore, a Finnish study [30] found that, although most patients had regained mostly normal HRQoL after 6 months post-injury, women had worse outcomes than men, as well as patients with mental disorders and especially major depressive disorder. It also discovered that the cohort women suffered more often from mental disorders and alcohol abuse and were more often unemployed. A history of psychiatric disease, especially depression, has shown to impose an important barrier to growth [31], which may hinder patients to regain a normal HRQoL. As mentioned above [27], about two-thirds of burn victims have a lifetime history of psychiatric disease, which increases the risk for further episodes post-burn. Therefore, screening for psychiatric disease in burn victims and focusing on psychological rehabilitation could be a possible way of assisting those patients to regain a normal HRQoL.

Another factor for a lower score in the SF-36 survey was third-degree burn injuries. They led to a lower result in the domains physical functioning, role limitation due to emotional functioning, vitality, emotional wellbeing and general health. Longer LOS showed a significant correlation with lower perceived physical functioning, role limitation due to physical functioning, pain and general health. Older patients reported a significantly lower physical functioning and scored lower in the role limitation due to the physical functioning domain. This was also noted in an Australian study [32], in which higher age had a negative impact on physical functioning and, to a lesser degree, on role limitations due to physical functioning, bodily pain, vitality and role emotional domains. Yet, these impairments should be relativized as a decline in bodily functions and arising comorbidities are a part of ageing. Moreover, Spronk et al. [28] discovered in a systematic review that the study situation is incoherent about the impact of age on HRQoL after burn injuries.

The %TBSA only had a significant correlation with the domain pain. Inhalation injury showed an insignificant correlation with general health, which might be due to the low incidence of inhalation injuries among the study population.

Against all expectations, no correlation between time since the injury or time since discharge and the self-reported HRQoL assessed with the SF-36 could be found. Furthermore, time since injury and time since discharge did not influence the answers to the burn-specific questions. Several studies [30,33,34,35], which had followed-up their patients between 6 months and 11.5 years post-burn, showed that HRQoL after burn injuries was affected in the short-term but increased and stabilized within the first few years. In the present study, the time since injury ranged from 17 to 107 months with a mean of 61.1 ± 25.4 months. As many participants were interviewed many months after that stabilizing period, this could be a possible reason for the dissociation of time since injury and the SF-36 domains.

### 4.3. Employment

Unexpectedly, a positive development in the employment status was found. Of the pre-burn injury employed male participants, 86% were employed upon interview and 62.9% of females. Only three previously employed participants were unemployed at the time of interview, and, compared to the time of the injury, unemployment had declined. At the time of the inquiry, there were only eight unemployed participants (−3.9%). As students and pupils had also been subsumed in the unemployment group, the decline in unemployment could be a sign that the patients who were in education were still able to start a career even after a burn injury.

Interestingly, employment status differed significantly between the female and male patients before the burn injury as well as after it. However, it did not have an influence on the return to work.

The strongest confounders for return to work were employment before the injury and, surprisingly, higher age. Moreover, a lower level of education showed a correlation with return to work. Having a job before the trauma could have been motivating for the patients in the process of recovery to return to their normal life. Orwelius et al. [8] found unemployment to have the strongest effect on HRQoL in their study population, and, on the other hand, Dyster-Aas et al. [6] discovered in their study cohort that patients who were working not only achieved better HRQoL but also physical and psychological health were significantly better than in those individuals who were not working. Another aspect that should not be left out is, as Goei et al. [36] pointed out, that absenteeism from work contributes to societal costs for burn victims.

### 4.4. Life Satisfaction

The domains autonomy, being needed, fulfillment and joy have shown to be fulfilled by the vast majority of the participants. For the domain being needed, a negative correlation with LOS could be found. A possible explanation could be that patients with a longer LOS usually sustained more severe and extensive injuries, which might have led to them needing more support from their environment instead of them being able to give support to others. This could also be a reason for the association with longer LOS and fulfillment. Only in the domain fulfillment with job or retirement was there a significant difference between the female and male participants, in which less women felt fulfilled in their job or retirement. For a possible explanation, further research is necessary. Unexpectedly, months since injury was found to be a negative confounder for joy, for which no explanation could be found.

When asked what participants would like to change, if they could, about a quarter of the patients stated the aesthetics of the wounds, a third would like to improve body functionality and 18.0% would change their mental wellbeing. The patients who wished to change their aesthetics had significantly longer LOS, were younger at the time of injury, female and had more likely sustained third-degree injuries. A reason could be that patients with longer LOS and third-degree injuries had evolved more scars, which changed their appearance. Moreover, physical appearance plays a more important role in the younger than in older age groups, and female patients have been seen to have a poorer opinion of their scars than male patients [37].

Better body functionality was wished for significantly more often by younger patients and by those who had sustained an injury of their hands as joints tend to lead to contractures and, thereby, can strongly impair functionality. Injury to the face or neck, interestingly, was negatively correlated with the wish for better functionality, which could be a sign that the healing of these injuries left little to no physical impairments.

At last, the wish for better mental wellbeing was significantly more frequent among the female participants, which aligns with the findings mentioned above, that significantly more female than male participants had a history of psychiatric disease. Moreover, a wish for better mental health was significantly associated with third-degree burn injuries, which could either be due to the trauma itself being a formative experience for the patient, with a lasting psychological burden, or due to necessary intensive treatment, which can be a strain for the patients.

### 4.5. Strengths and Limitations

As the participants were accompanied through the questions by the interviewer, upcoming unclear questions could be explained immediately and no questions were left blank.

What has to be taken into consideration is that the study was conducted during the SARS-CoV-19 pandemic, which put a strain on most peoples’ lives as many struggled with illness, social withdrawal, isolation and altered employment situations.

Moreover, the study was conducted retrospectively and had no baseline data from before the accident nor from an earlier rehabilitation point. This means that the study was not able to assess how HRQoL developed and whether it returned to the individual pre-injury value.

Furthermore, most of the participants suffered minor burns and had a relatively short LOS, which might have been a less formative experience with little scarring and other complications. It is possible that the patients that could not be reached or were not willing to participate had sustained more extensive and severe injuries.

Finally, although the SF-36 was conceptualized for paper–pencil, as well as for telephone administration [38], some patients had difficulties following the questions and the various response options. This could have impacted the given answers. Another influence on the accuracy of the answers could be the lack of anonymity during the interview as the patients had to answer to the interviewer instead of filling out the form anonymously at their own pace.

## 5. Conclusions

First of all, our study showed that female patients’ outcomes of HRQoL and life satisfaction were significantly lower than their male counterparts. Moreover, burn injuries do not seem to have a negative impact on return to work in our study collective as most of the patients returned to their work and unemployment had also declined. All in all, life satisfaction seems to be fulfilled by the vast majority of the participants, which, together with the employment trend, is a sign that most of the participants had reintegrated well into society and the majority of the patients stated to be content with their aesthetical, functional and mental outcomes after burn injury.

Further research should focus on reasons for this gender discrepancy and possible target points. This could provide useful information for optimized follow-up treatment and rehabilitation.

At last, it must be considered that these findings depict the outcomes for only about a third of the patients treated between 2012 and 2019, and there is the risk of bias in that patients with worse outcomes were not reached or were unwilling to participate.

## Figures and Tables

**Table 1:** Complementary questions.

Employment, Level of Education and Life Satisfaction		
	Yes	No
Employment before burn injury		
a Self-employed	〇	〇
b Employee, civil servant	〇	〇
c Worker	〇	〇
d Retiree	〇	〇
e Unemployed	〇	〇
Employment after burn injury		
a Self-employed	〇	〇
b Employee, civil servant	〇	〇
c Worker	〇	〇
d Retiree	〇	〇
e Unemployed	〇	〇
If employed: Change in employment		
a No change (same job and hours)	〇	〇
b Same job and less hours	〇	〇
c Different job and same hours	〇	〇
d Different job and less hours	〇	〇
Highest level of education		
a Middle school	〇	〇
b Apprenticeship	〇	〇
c General qualification for university entrance	〇	〇
d University degree	〇	〇
Description of current life situation		
a Leading an autonomous and self-determined life	〇	〇
b Being needed in everyday life	〇	〇
c Fulfilling work/retirement	〇	〇
d Enjoyable leisure activities	〇	〇
e Desire to change something, if possible	〇	〇
i Appearance (e.g., unsightly scars)	〇	〇
ii Body functionality (e.g., dry skin, mobility)	〇	〇
iii Mental wellbeing (e.g., persisting sleep disorders or anxiety)	〇	〇

**Table 2:** Demography of participants.

Demography of Participants				
	Female	Male	Total	*p*-Value
Number of patients (%)	35 (27.3%)	93 (72.7%)	128 (100%)	
Age at injury				
Mean (SD)	41.2 (SD 16.3)	39.5 (SD 15.6)	40.0 (SD 15.7)	0.594 *
Age at inquiry				
Mean (SD)	46.1 (SD 16.4)	44.7 (SD 16.2)	45.1 (SD 16.2)	0.662 *
Pre-existing conditions				
Diabetes mellitus	1 (2.9%)	1 (1.1%)	2 (1.6%)	0.469 **
Arterial hypertension	8 (22.9%)	11 (11.8%)	19 (14.8%)	0.118 **
Ischemic heart disease	3 (8.6%)	2 (2.2%)	5 (3.9%)	0.095 **
Cerebrovascular disease	1 (2.9%)	1 (1.1%)	2 (1.6%)	0.469 **
Peripheral vascular disease	1 (2.9%)	0	1 (0.8%)	0.102 **
Neurologic disease	3 (8.6%)	2 (2.2%)	5 (3.9%)	0.095 **
History of psychiatric disease	6 (17.1%)	2 (2.2%)	8 (6.3%)	0.002 **
History of substance abuse	2 (5.7%)	0	2 (1.6%)	0.020 **
History of alcohol abuse	3 (8.6%)	1 (1.1%)	4 (3.1%)	0.030 **
Documented smoker	3 (8.6%)	10 (10.8%)	13 (10.2%)	0.716 **
Other comorbidities	2 (5.7%)	4 (4.3%)	6 (4.7%)	0.736 **

* *t*-test, ** Pearson’s chi-square.

**Table 3:** Injury characteristics of participants.

Injury Characteristics				
	Female	Male	Total	*p*-Value
Injury mechanism				0.292 *
Flame (%)	15 (42.9%)	56 (60.2%)	71 (55.5%)	
Scald (%)	14 (40.0%)	26 (28.0%)	40 (31.3%)	
Contact burn (%)	3 (8.6%)	4 (4.3%)	7 (5.5%)	
Chemical burn (%)	3 (8.6%)	4 (4.3%)	7 (5.5%)	
Electric burn (%)	0	3 (3.2%)	3 (2.3%)	
Circumstances				0.011 *
Work-related	8 (22.9%)	43 (46.2%)	51 (39.5%)	
Household	21 (60.0%)	47 (50.5%)	68 (53.1%)	
Traffic accident	2 (5.7%)	3 (3.2%)	5 (3.9%)	
Self-inflicted	1 (2.9%)	0	1 (0.8%)	
Assault	1 (2.9%)	0	1 (0.8%)	
Other	2 (5.7%)	0	2 (1.6%)	
%TBSA				
Mean (SD)	5.6 (SD 7.3)	10.6 (SD 11.8)	9.2 (SD 11.0)	0.005 **
No. of patients with third-degree burns (%)	15 (42.9%)	51 (54.8%)	66 (51.6%)	0.227 *
Affected body area				
Upper extremity	17 (48.6%)	65 (69.9%)	82 (64.1%)	
Lower extremity	23 (65.7%)	45 (48.4%)	68 (53.1%)	
Back	4 (11.4%)	13 (14.0%)	17 (13.3%)	
Ant. thorax/abdomen	8 (22.9%)	22 (23.7%)	30 (23.4%)	
Genitals	1 (2.9%)	5 (5.4%)	6 (4.7%)	0.548 *
Hands	9 (25.7%)	44 (47.3%)	53 (41.4%)	0.024 *
Face/neck	11 (31.4%)	52 (55.9%)	63 (49.2%)	0.014 *
Inhalation injury	0	5 (5.4%)	5 (3.9%)	0.162 *

* Pearson’s chi-square, ** *t*-test.

**Table 4:** Survey outcome of SF-36 domains.

Survey Outcome				
	Female	Male	Total	*p*-Value *
Months since injury (mean, SD)	58.9 (SD 26.5)	62.0 (SD 25.1)	61.1 (SD 25.4)	0.538
SF-36 scale scores (mean, SD)				
Physical functioning	77.3 (SD 29.0)	91.2 (SD 16.3)	87.3 (SD 21.4)	0.010
Role—physical	62.1 (SD 47.5)	83.3 (SD 35.09)	77.5 (SD 40.0)	0.020
Role—emotional	55.2 (SD 49.8)	89.6 (SD 30.3)	80.2 (SD 40.0)	0.000
Vitality	51.9 (SD 21.0)	64.3 (SD 18.1)	80.9 (SD 19.7)	0.003
Mental health	66.6 (SD 21.9)	81.3 (SD 13.7)	77.3 (SD 17.5)	0.001
Social functioning	82.9 (SD 33.9)	95.0 (SD 17.9)	91.7 (SD 23.9)	0.050
Bodily Pain	64.8 (SD 40.3)	79.2 (SD 30.3)	75.2 (SD 33.8)	0.061
General health	67.6 (SD 29.8)	86.0 (SD 20.8)	81.0 (SD 24.9)	0.002

* *t*-test.

**Table 5:** Employment status of participants pre- and post-burn-injury.

Employment				
		Female	Male	Total
Pre-burn *	Self-employed	2 (5.7%)	10 (10.8%)	12 (9.4%)
	Employee/civil servant	12 (34.3%)	35 (37.6%)	47 (36.7%)
	Worker	4 (11.4%)	32 (34.4%)	36 (28.1%)
	Retiree	9 (25.7%)	11 (11.8%)	20 (15.6%)
	Unemployed	8 (22.9%)	5 (5.4%)	13 (10.2%)
Post-burn **	Self-employed	2 (5.7%)	11 (11.8%)	13 (10.2%)
	Employee/civil servant	14 (40%)	34 (36.6%)	48 (37.5%)
	Worker	2 (5.7%)	25 (26.9%)	27 (21.1%)
	Retiree	13 (37.1%)	19 (20.4%)	32 (25.0%)
	Unemployed	4 (11.4%)	4 (4.3%)	8 (6.3%)
Change in employment	Same job and hours ***	20 (57.1%)	67 (72.0%)	87 (68.0%)
	Change in job ***	1 (2.9%)	7 (7.5%)	8 (6.3%)
	Change in hours ***	0	2 (2.2%)	2 (1.6%)
	Change in job and hours ***	1 (2.9%)	4 (4.3%)	5 (3.9%)
	Got a job	6 (17.1%)	2 (2.2%)	8 (6.3%)
	Retired	4 (11.4%)	8 (8.6%)	12 (9.4%)
	Lost job	2 (5.7%)	1 (1.1%)	3 (2.3%)
	Still unemployed	1 (2.9%)	2 (2.2.%)	3 (2.3%)

* Pearson’s chi-square *p* = 0.003, ** Pearson’s chi-square *p* = 0.023, *** Spearman correlation *p* = 0.898.

**Table 6:** Outcome of complementary questions considering life satisfaction.

Social Reintegration				
	Female	Male	Total	*p*-Value *
Autonomous, self-determined life	33 (94.3%)	91 (97.8%)	124 (96.9%)	0.302
Being needed in everyday life	33 (94.3%)	89 (95.7%)	122 (95.3%)	0.736
Fulfilling job/retirement	27 (77.1%)	84 (90.3%)	111 (86.7%)	0.050
Enjoyable leisure activities	30 (85.7%)	88 (94.6%)	118 (92.2%)	0.094
Would change aesthetics	11 (31.4%)	20 (21.5%)	31 (24.2%)	0.243
Would change body functionality	14 (40.0%)	29 (31.2%)	43 (33.6%)	0.347
Would change mental wellbeing	11 (31.4%)	12 (12.9%)	23 (18.0%)	0.015

* Pearson’s chi-square.

## Data Availability

Not applicable.

## References

[1] Pereira C., Murphy K., Herndon D. (2004). Outcome measures in burn care: Is mortality dead?. Burns.

[2] Jeschke M.G., van Baar M.E., Choudhry M.A., Chung K.K., Gibran N.S., Logsetty S. (2020). Burn injury. Nat. Rev. Dis. Primers.

[3] Kamolz L.P., Herndon D.N., Jeschke M.G. (2009). Verbrennungen: Diagnose, Therapie und Rehabilitation des thermischen Traumas.

[4] Wasiak J., Lee S.J., Paul E., Mahar P., Pfitzer B., Spinks A., Cleland H., Gabbe B. (2014). Predictors of health status and health-related quality of life 12 months after severe burn. Burns.

[5] Gauffin E., Öster C., Sjöberg F., Gerdin B., Ekselius L. (2016). Health-related quality of life (EQ-5D) early after injury predicts long-term pain after burn. Burns.

[6] Dyster-Aas J., Kildal M., Willebrand M. (2007). Return to work and health-related quality of life after burn injury. J. Rehabil. Med..

[7] Öster C., Kildal M., Ekselius L. (2010). Return to work after burn injury: Burn-injured individuals’ perception of barriers and facilitators. J. Burn Care Res..

[8] Orwelius L., Willebrand M., Gerdin B., Ekselius L., Fredrikson M., Sjöberg F. (2013). Long term health-related quality of life after burns is strongly dependent on pre-existing disease and psychosocial issues and less due to the burn itself. Burns.

[9] Mason S.T., Esselman P., Fraser R., Schomer K., Truitt A., Johnson K. (2012). Return to work after burn injury: A systematic review. J. Burn Care Res..

[10] Palmu R., Partonen T., Suominen K., Vuola J., Isometsä E. (2015). Return to work six months after burn: A prospective study at the Helsinki Burn Center. Burns.

[11] Quinn T., Wasiak J., Cleland H. (2010). An examination of factors that affect return to work following burns: A systematic review of the literature. Burns.

[12] Tolentino-Bazán K., Chavez-Heres T., Morales-García M., Macías-Hernández S.I., Ramírez-Ramírez A.C., Velázquez-Bustamante A.E., Rhoades-Torres G.M., Velez-Palafox M. (2021). Predictive Factors for Returning to Work in Burn Adult Patients That Were Working Before Their Injury. J. Burn Care Res..

[13] Öster C., Ekselius L. (2011). Return to work after burn-A prospective study. Burns.

[14] Esselman P.C., Wiechman Askay S., Carrougher G.J., Lezotte D.C., Holavanahalli R.K., Magyar-Russell G., Fauerbach J.A., Engrav L.H. (2007). Barriers to return to work after burn injuries. Arch. Phys. Med. Rehabil..

[15] Goverman J., Mathews K., Nadler D., Henderson E., McMullen K., Herndon D., Meyer W., Fauerbach J., Wiechman S., Carrougher G. (2016). Satisfaction with life after burn: A Burn Model System National Database Study. Burns.

[16] Amtmann D., Bocell F.D., McMullen K., Bamer A.M., Johnson K.L., Wiechman S.A., Schneider J.C. (2020). Satisfaction With Life Over Time in People With Burn Injury: A National Institute on Disability, Independent Living, and Rehabilitation Research Burn Model System Study. Arch. Phys. Med. Rehabil..

[17] Amtmann D., Bocell F.D., Bamer A., Heinemann A.W., Hoffman J.M., Juengst S.B., Rosenberg M., Schneider J.C., Wiechman S., McMullen K. (2019). Psychometric Properties of the Satisfaction With Life Scale in People With Traumatic Brain, Spinal Cord, or Burn Injury: A National Institute on Disability, Independent Living, and Rehabilitation Research Model System Study. Assessment.

[18] Edgar D., Dawson A., Hankey G., Phillips M., Wood F. (2010). Demonstration of the validity of the SF-36 for measurement of the temporal recovery of quality of life outcomes in burns survivors. Burns.

[19] Bullinger M., Kirchberger I., Ware J. (1995). Der deutsche SF-36 Health Survey Übersetzung und psychometrische Testung eines krankheitsübergreifenden Instruments zur Erfassung der gesundheitsbezogenen Lebensqualität. Z. Gesundh..

[20] Morfeld M., Bullinger M. (2008). Der SF36 Health Survey zur Erhebung und Dokumentation gesundheitsbezogener Lebensqualität. Phys. Med. Rehabil. Kurortmed..

[21] SF-36 Fragebogen Auswertung und Inhalt (Short Form 36). Heartbeat. https://heartbeat-med.com/de/wiki/sf-36-fragebogen/#.

[22] Gojowy D., Kauke M., Ohmann T., Homann H.-H., Mannil L. (2019). Early and late-recorded predictors of health-related quality of life of burn patients on long-term follow-up. Burns.

[23] Ellert U., Kurth B.-M. (2004). Methodische Betrachtungen zu den Summenscores des SF-36 anhand der erwachsenen bundesdeutschen Bevölkerung. Bundesgesundheitsblatt-Gesundheitsforschung-Gesundheitsschutz.

[24] Smolle C., Cambiaso-Daniel J., Forbes A.A., Wurzer P., Hundeshagen G., Branski L.K., Huss F., Kamolz L. (2017). Recent trends in burn epidemiology worldwide: A systematic review. Burns.

[25] Peck M.D. (2011). Epidemiology of burns throughout the world. Part I: Distribution and risk factors. Burns.

[26] Brusselaers N., Monstrey S., Vogelaers D., Hoste E., Blot S. (2010). Severe burn injury in europe: A systematic review of the incidence, etiology, morbidity, and mortality. Crit. Care.

[27] Dyster-Aas J., Willebrand M., Wikehult B., Gerdin B., Ekselius L. (2008). Major Depression and Posttraumatic Stress Disorder Symptoms Following Severe Burn Injury in Relation to Lifetime Psychiatric Morbidity. J. Trauma.

[28] Spronk I., Legemate C.M., Dokter J., van Loey N.E.E., van Baar M.E., Polinder S. (2018). Predictors of health-related quality of life after burn injuries: A systematic review. Crit. Care.

[29] Spronk I., van Loey N.E.E., Sewalt C., Nieboer D., Renneberg B., Moi A.L., Oster C., Orwelius L., Van Baar M.E., Polinder S. (2020). Recovery of health-related quality of life after burn injuries: An individual participant data meta-analysis. PLoS ONE.

[30] Palmu R., Partonen T., Suominen K., Saarni S.I., Vuola J., Isometsä E. (2015). Health-related quality of life 6 months after burns among hospitalized patients: Predictive importance of mental disorders and burn severity. Burns.

[31] Martin L., Byrnes M., Bulsara M.K., McGarry S., Rea S., Wood F. (2017). Quality of life and posttraumatic growth after adult burn: A prospective, longitudinal study. Burns.

[32] Edgar D.W., Homer L., Phillips M., Gurfinkel R., Rea S., Wood F.M. (2013). The influence of advancing age on quality of life and rate of recovery after treatment for burn. Burns.

[33] Renneberg B., Ripper S., Schulze J., Seehausen A., Weiler M., Wind G., Liedl A. (2014). Quality of life and predictors of long-term outcome after severe burn injury. J. Behav. Med..

[34] Moi A.L., Haugsmyr E., Heisterkamp H. (2016). Long-Term Study Of Health And Quality Of Life After Burn Injury. Ann. Burns Fire Disasters.

[35] Fauerbach J.A., Lezotte D.C., Hills R.A., Cromes F.G., Kowalske K.J., de Lateur B.J., Goodwin C.W., Blakeney P., Herndon D.N., Wiechman S.A. (2005). Burden of Burn: A Norm-Based Inquiry into the Influence of Burn Size and Distress on Recovery of Physical and Psychosocial Function. J. Burn Care Rehabil..

[36] Goei H., Hop M.J., van der Vlies C.H., Nieuwenhuis M.K., Polinder S., Middelkoop E., van Baar M.E. (2016). Return to work after specialised burn care: A two-year prospective follow-up study of the prevalence, predictors and related costs. Injury.

[37] Spronk I., Polinder S., Haagsma J.A., Nieuwenhuis M., Pijpe A., van der Vlies C., Middelkoop E., van Baar M. (2019). Patient-reported scar quality of adults after burn injuries: A five-year multicenter follow-up study. Wound Repair Regen..

[38] Ware J.E., Sherbourne C.D. (1992). The MOS 36-item short-form health survey (SF-36). I. Conceptual framework and item selection. Med. Care.

